# Poly(I:C) Induces Antiviral Immune Responses in Japanese Flounder (*Paralichthys olivaceus*) That Require TLR3 and MDA5 and Is Negatively Regulated by Myd88

**DOI:** 10.1371/journal.pone.0112918

**Published:** 2014-11-13

**Authors:** Zhi-xia Zhou, Bao-cun Zhang, Li Sun

**Affiliations:** 1 Key Laboratory of Experimental Marine Biology, Institute of Oceanology, Chinese Academy of Sciences, Qingdao, China; 2 University of Chinese Academy of Sciences, Beijing, China; 3 Collaborative Innovation Center of Deep Sea Biology, Zhejiang University, Hangzhou, China; Friedrich-Alexander-University Erlangen, Germany

## Abstract

Polyinosinic:polycytidylic acid (poly(I:C)) is a ligand of toll-like receptor (TLR) 3 that has been used as an immunostimulant in humans and mice against viral diseases based on its ability to enhance innate and adapt immunity. Antiviral effect of poly(I:C) has also been observed in teleost, however, the underling mechanism is not clear. In this study, we investigated the potential and signaling mechanism of poly(I:C) as an antiviral agent in a model of Japanese flounder (*Paralichthys olivaceus*) infected with megalocytivirus. We found that poly(I:C) exhibited strong antiviral activity and enhanced activation of head kidney macrophages and peripheral blood leukocytes. *In vivo* studies showed that (i) TLR3 as well as MDA5 knockdown reduced poly(I:C)-mediated immune response and antiviral activity to significant extents; (ii) when Myd88 was overexpressed in flounder, poly(I:C)-mediated antiviral activity was significantly decreased; (iii) when Myd88 was inactivated, the antiviral effect of poly(I:C) was significantly increased. Cellular study showed that (i) the NF-κB activity induced by poly(I:C) was upregulated in Myd88-overexpressing cells and unaffected in Myd88-inactivated cells; (ii) Myd88 overexpression inhibited and upregulated the expression of poly(I:C)-induced antiviral genes and inflammatory genes respectively; (iii) Myd88 inactivation enhanced the expression of the antiviral genes induced by poly(I:C). Taken together, these results indicate that poly(I:C) is an immunostimulant with antiviral potential, and that the immune response of poly(I:C) requires TLR3 and MDA5 and is negatively regulated by Myd88 in a manner not involving NK-κB. These results provide insights to the working mechanism of poly(I:C), TLR3, and Myd88 in fish.

## Introduction

Polyinosinic:polycytidylic acid (poly(I:C)) is a structural analogue of double-stranded RNA (dsRNA). It has been used widely in the study of immune responses associated with viral infection. Poly(I:C) exerts its biological effect by interacting with the toll-like receptor (TLR) family member TLR3, which is broadly expressed and well conserved among vertebrates. In mammals, TLR3 expression has been confirmed in immune and non-immune cells, such as dendritic cells, B cells, macrophages, epithelial cells, endothelial cells, fibroblasts, keratinocytes, and tumor cells [1]–[4]. In contrast to other TLRs, TLR3 signaling occurs via TIR-domain-containing adapter-inducing interferon-β (TRIF)-dependent pathways and does not require myeloid differentiation primary response 88 (Myd88)-dependent pathways [5]. Upon binding to dsRNA, TLR3 signaling is activated, which leads to three major outcomes in inflammation and innate immunity: (i) development of antiviral response mediated by activation of IFN regulatory transcription factor (IRF) 3 and IRF7 and by production of type I IFN [6]; (ii) generation of a pro-inflammatory environment by the activation of pro-inflammatory and pro-survival transcription factors, nuclear factor-κB (NF-κB), and activator protein 1 (AP-1) [7]; (iii) induction of cytopathic effect or cell death in a caspase-8-dependent fashion via receptor interacting protein 1 (RIP1) [2]. In addition, TLR3 signaling also modulates adaptive immune responses, e.g. enhancing the cytotoxic activity of γδT cells and mediating cross-priming of cytotoxic T lymphocytes (CTLs) against cell-associated antigens in CD8^+^ dendritic cells (DCs) [8], [9]. TLR3 signaling can also upregulate the expression of positive and negative co-stimulatory molecules on DCs and influence the magnitude of CD8^+^ T cell responses [10].

In teleost, orthologs of mammalian TLR3 have been identified in a number of species, i.e. Japanese flounder (*Paralichthys olivaceus*) [11], catfish (*Ictalurus punctatus*) [12], fugu (*Takifugu rubripes*) [13], rainbow trout (*Oncorhynchus mykiss*) [14], zebrafish (*Danio rerio*) [15], and Atlantic salmon (*Salmo salar*) [16]. In some of these species, poly(I:C) is known to exhibit an inhibitory effect on the infection of fish viruses such as hemorrhagic septicemia virus [17], infectious hematopoietic necrosis [18], infectious salmon anaemia virus [19], haematopoietic necrosis virus [20], infectious pancreatic necrosis virus [21], and channel catfish virus [22]. However, the working mechanism of poly(I:C) in teleost is unclear, and very little is known about the relationship between poly(I:C)-induced antiviral effect and TLR3 signaling.

Japanese flounder is an economically important fish species farmed widely in the world. In the present study, with flounder as a host model and megalocytivirus as the infectious agent, we investigated the effect of poly(I:C) on antiviral immunity and the signaling pathway that mediates this effect. We found that poly(I:C) exhibited apparent immunostimulatory properties, which are dependent on TLR3 and negatively regulated by Myd88.

## Materials and Methods

### Ethics statement

Experiments involving live animals were conducted in accordance with the “Regulations for the Administration of Affairs Concerning Experimental Animals” promulgated by the State Science and Technology Commission of Shandong Province. The study was approved by the ethics committee of Institute of Oceanology, Chinese Academy of Sciences.

### Fish

Clinically healthy Japanese flounder (*Paralichthys olivaceus*) were purchased from a local fish farm and acclimatized in the laboratory for two weeks before experimental manipulation. Fish were fed daily with commercial dry pellets and maintained at 22°C in aerated seawater. Before experiments, fish (5% of stock) were randomly sampled for the examination of bacteria or megalocytivirus in blood, liver, kidney, and spleen. No bacteria or virus were detected from the sampled fish. For tissue collection, fish were euthanized with tricaine methanesulfonate (Sigma, St. Louis, MO, USA) as described previously [23].

### Cell line

FG-9307, a cell line established from Japanese flounder gill cells [24], was cultured as reported previously [24] at 22°C in L-15 medium (Thermo Scientific HyClone, Beijing, China) supplemented with 100 U/ml penicillin (Sangon, Shanghai, China), 100 µg/ml streptomycin (Sangon, Shanghai, China) and 10% fetal bovine serum (FBS) (Thermo Scientific HyClone, Beijing, China).

### Effect of poly(I:C) on viral infection

Japanese flounder (average 10.3 g) were divided randomly into two groups (12 fish/group) and administered intramuscularly (i.m.) with poly(I:C) (InvivoGen, San Diego, CA, USA) at 20 µg/fish or with phosphate buffered saline (PBS) as a control. At 24 h post-administration, the fish were challenged intraperitoneally (i.p.) with 100 µl of megalocytivirus that had been suspended in PBS to 5×10^6^ copies/ml. At 3 d, 5 d, and 7 d post-challenge, kidney, spleen, and liver from the fish (three fish/time point) were collected, and the viral amounts in the tissues were determined by absolute quantitative real time PCR as reported previously [25].

### Preparation of head kidney macrophages (HKM) and peripheral blood leukocytes (PBL)

Head kidney was removed from Japanese flounder, washed three times with PBS, and passed through a sterile metal mesh. The cells were resuspended in L-15 medium (Thermo Scientific HyClone, Beijing, China) and placed onto a 34/51% Percoll (Solarbio, Beijing, China) gradient. After centrifugation at 400×*g* for 30 min, the cells at the 34/51% interface were recovered, washed twice with PBS, and resuspended in L-15 medium containing 10% FBS (Thermo Scientific HyClone, Beijing, China), 100 U/ml penicillin (Sangon, Shanghai, China), and 100 µg/ml streptomycin (Sangon, Shanghai, China). The cells were distributed into 96-well tissue culture plates (∼1×10^5^ cells/well) and incubated at 22°C for 2 h. Non-adherent cells were washed off after the incubation. PBL were isolated from Japanese flounder as reported previously [26].

### Effect of poly(I:C) on the respiratory burst of HKM

Japanese flounder were divided randomly into four groups (20 fish/group) and administered i.m. with poly(I:C) at different doses (4 µg/fish, 20 µg/fish, and 100 µg/fish) or with PBS as a control. At 1 d, 3 d, 5 d, and 7 d post-poly(I:C) administration, HKM were prepared from the fish (four fish/time point) as described above and used for respiratory burst assay as reported previously [27].

### Determination of the cytotoxicity of PBL by lactate dehydrogenase (LDH) assay

Cytotoxicity was performed with the LDH kit (Roche Applied Science, Indianapolis, IN, USA) according to the manufacturer's instructions. Briefly, for effector cell preparation, Japanese flounder were injected i.m. with 20 µg poly(I:C) or PBS (control). At 5 d post-injection, PBL were prepared as described above and designated as effector cells. For target cell preparation, flounder were infected with megalocytivirus as described above. At 5 d post-infection (dpi), PBL were prepared as described above and designated as target cells. For LDH assay, the target PBL cells were distributed into a 96-well U-bottomed plate (1×10^5^ cells/well), and the effector PBL were added to the plate to the ratios (effector∶target) of 1∶1, 2∶1, 4∶1, and 8∶1. The plate was incubated at 22°C for 24 h and centrifuged at 250 *g* for 10 min. Aliquots (100 µl/well) were transferred to a fresh 96-well flat-bottom plate. To determine the LDH activity in the culture supernatant, an equal volume of freshly prepared reaction mixture (from the LDH kit) was added to the plate. The plate was incubated in the dark at room temperature for 30 min, and absorbance at 492 nm was measured. Cytotoxicity was calculated using the following formula: cytotoxicity (%) = {[*A*_492_ (effector/target)−*A*_492_ (effector control)−*A*_492_ (target control)]/[*A*_492_ (target maximum)−*A*_492_ (target control)]}×100%.

### Quantitative real-time reverse transcription-PCR (qRT-PCR)

Total RNA was extracted from cells or tissues with the RNAprep Tissue Kit (Tiangen, Beijing, China). One microgram of total RNA was used for cDNA synthesis with M-MLV reverse transcriptase (Invitrogen, Carlsbad, CA, USA). qRT-PCR was carried out in an Eppendorf Mastercycler (Eppendorf, Hamburg, Germany) using the SYBR ExScript qRT-PCR Kit (Takara, Dalian, China) as described previously [28]. Melting curve analysis of amplification products was performed at the end of each PCR to confirm that only one PCR product was amplified and detected. The expression level of the target gene was analyzed using comparative threshold cycle method with beta-actin (for samples without viral infection) or elongation factor-1-alpha (EF1 alpha) (for samples from virus-infected fish) as the internal reference [28], [29]. All data are given in terms of mRNA levels relative to that of the internal reference and expressed as means plus or minus standard errors of the means (SE).

### RNA interference

#### (i) *In vitro* RNA interference

Three small interfering (si) RNA targeting different regions of TLR3 were synthesized by Ribobio (Guangzhou, China) and named siTLR3, siR2, and siR3. The sequences of siTLR3, siR2, and siR3 are 5′-GCUGGAGGAUUCAGUUCUAdTdT-3′, 5′-GCUGCAGAAAGGAAAUCUAdTdT-3′, and 5′-GGUUGAAUAUGGCGAGUAAdTdT-3′ respectively. The control siRNA (named siRC) was provided by the company (sequence not disclosed by the company). *In vitro* transfection of siRNAs was carried out using Lipofectamine RNAiMAX (Invitrogen, Carlsbad, CA). To examine transfection efficiency, FG cells were transfected with fluorescent-labeled siRNAs (final concentration 100 nM) for 24 h according to the manufacturer's instructions. Transfection efficiency was determined with the riboMONITOR Kit (Ribobio, Guangzhou, China) as described previously [27]. To examine the potential of the three siRNAs to block TLR3 expression, FG cells were transfected with each of the siRNA or siRC for 24 h, and the expression of TLR3 was determined by qRT-PCR as described above. For melanoma differentiation associated gene 5 (MDA5) knockdown, three siRNA targeting MDA5 were synthesized by Ribobio (Guangzhou, China) and named siMDA5 (5′- GGAGAGAUCCAACAAAGAAdTdT -3′), siR6 (5′-GCAGAGUCUUGCAGGGAAUdTdT-3′), and siR7 (5′-GCCAUUCAUGCUCAUGCAAdTdT-3′). The control siRNA (named siRC2) was provided by the company (sequence not disclosed by the company). FG cell transfection with siMDA5, siR6, and siR7 and examination of gene expression were performed as above.

#### (ii) *In vivo* RNA interference

For *in vivo* knockdown of TLR3 expression in flounder, siRNA was dissolved in 0.9% NaCl and mixed with Lipofectamine RNAiMAX according to manufacturer's instructions. Flounder were divided randomly into four equal-sized groups named A to D. Groups A and B were injected i.p. with siTLR3 and siRC (10 µg/fish) respectively, while groups C and D were injected with PBS. At 24 h post-injection, groups A, B, and C were injected i.m. with 20 µg poly(I:C), and group D was injected similarly with PBS (control). At 24 h post-injection, three fish were removed from each group, and the spleen of the fish was used for qRT-PCR analysis of the expression of TLR3, interleukin (IL)-1β, IL-6, tumor necrosis factor alpha (TNF-α), type I interferon (IFN-I), Mx and IFN-stimulated gene (ISG) 56 as described above.

### Plasmid construction

To construct pCNMyd88, which expresses constitutively a His-tagged flounder Myd88 under the human cytomegalovirus immediate-early promoter, the open reading frame of Myd88 was amplified by PCR with primers Myd88F1 (5′-GATATCGCCACCATGGCCTGTGCAGACT-3′, underlined, EcoRV site) and Myd88R1 (5′-GCGCGATATCATGGCCTGTGCAGACTTTG-3′, underlined, EcoRV site); the PCR products were ligated with the T-A cloning vector pBS-T (Tiangen, Beijing, China), and the recombinant plasmid was digested with EcoRV to retrieve the Myd88 fragment, which was inserted into pCN3 [30] at the EcoRV site.

### Overexpression of Myd88 in Japanese flounder

pCNMyd88 and pCN3 were diluted in PBS to 200 µg/ml. Japanese flounder were divided randomly into three groups (6 fish/group) and injected i.m. with 100 µl of pCNMyd88, pCN3, or PBS. Muscle, kidney, liver, and spleen were taken from the fish at 2 d post-plasmid administration. To examine the presence of plasmid in the tissues, DNA was extracted from the tissues with TIANamp DNA Kit (Tiangen, Beijing, China) and used for PCR with the pCNMyd88-specific primers Myd88F1 (as above) and CNR1 (5′-TGCGGGCCTCTTCGCTATT-3′) and the pCN3-specific primers CNF1 (5′-CTTGCGTTTCTGATAGGCACCTA-3′) and CNR1. To examine the expression of plasmid-derived Myd88 in the tissues, total RNA was extracted from the tissues and used for qRT-PCR with primers Myd88F1 and HisR (5′-GTGGTGGTGGTGGTGGTG-3′), which amplify the Myd88 sequence in pCNMyd88.

### Effect of Myd88 overexpression on poly(I:C)-induced antiviral activity

Flounder were injected i.m. with pCNMyd88, pCN3, or PBS as described above. At 2 d post-plasmid injection, the fish were injected i.m. with 20 µg poly(I:C). At 1 d post-poly(I:C) injection, the fish were challenged with megalocytivirus as described above. At 3 d, 5 d, and 7 d post-challenge, kidney and spleen from the fish (three fish/time point) were collected, and the viral amounts in the tissues were determined by absolute qRT-PCR as described above.

### Effect of Myd88 inactivation on poly(I:C)-induced antiviral activity

Pepinh-MYD (RQIKIWFQNRRMKWKK-RDVLPGTCVNS-NH2) is a 26-amino acid peptide that blocks Myd88 signaling (InvivoGen, San Diego, CA, USA). Pepinh-Control (RQIKIWFQNRRMKWKK-SLHGRGDPMEAFII-NH2) is a control peptide provided by the company. To examine the effect of Myd88 inactivation on poly(I:C)-induced antiviral activity, flounder were divided randomly into six equal-sized groups named A to F. Groups A and B, C and D, and E and F were administered via i.p. injection with 50 µM Pepinh-MYD, 50 µM Pepinh-Control, and PBS respectively. At 6 h post-administration, groups A, C, and E were injected i.m. with 20 µg poly(I:C), while the other three groups were injected with PBS. At 1 d post-poly(I:C) injection, the fish were challenged with megalocytivirus as described above. At 3 d, 5 d, and 7 d post-challenge, kidney and spleen from the fish (three fish/time point) were collected, and the viral amounts in the tissues were determined as above.

### Effect of Myd88 on the expression of immune genes in FG cells

FG cells were distributed into 12-well culture plates (4×10^5^ cells/well) in Opti-MEMI Reduced-Serum Medium (GIBCO) without FBS. For Myd88 overexpression, the cells were transfected with pCNMyd88 and pCN3 using Lipofectamine LTX and PLUS (Invitrogen, Carlsbad, CA, USA) according to the instructions of the manufacturer. Poly(I:C) was then added to the plates to the final concentration of 20 µg/ml, and the plates were incubated at 22°C for 24 h. The cells were then used for qRT-PCR analysis of the expression of IL-1β, IL-6, TNF-α, IFN I, IFN-γ, Mx, and ISG56 as above. For Myd88 inactivation, Pepinh-MYD and Pepinh-Control were added to the cells to the concentration of 50 µM, and the cells were incubated at 22°C for 6 h. After incubation, the cells were treated with poly(I:C) and examined for gene expression as above.

### Luciferase reporter assay

Myd88 overexpression and inactivation in FG cells were performed as described above. For luciferase assay, the cells were co-transfected with the firefly NF-κB–specific luciferase reporter vector pNFκB-Met-Luc2 (Clontech, Mountain View, CA, USA). Transfection efficiency was monitored by co-transfection with the pSEAP2 control vector (Clontech, Mountain View, CA, USA), which constitutively expresses the human secreted enhanced alkaline phosphatase (SEAP) [31]. Poly(I:C) was added to the plates to the final concentration of 20 µg/ml, and the cells were incubated at 22°C for 24 h. The culture medium of the transfectants were then analyzed for luciferase activity and SEAP activity using Luciferase Assay Kit and the Great EscAPe SEAP Chemiluminescence Detection Kit (Clontech, Mountain View, CA, USA) respectively.

### Statistical analysis

All experiments were performed three times, and statistical analyses were carried out with SPSS 17.0 software (SPSS Inc., Chicago, IL, USA). Analysis of variance (ANOVA) was used for all analyses. In all cases, the significance level was defined as *P*<0.05

## Results

### Antiviral effect of poly(I:C) in Japanese flounder

**Figure 1:**
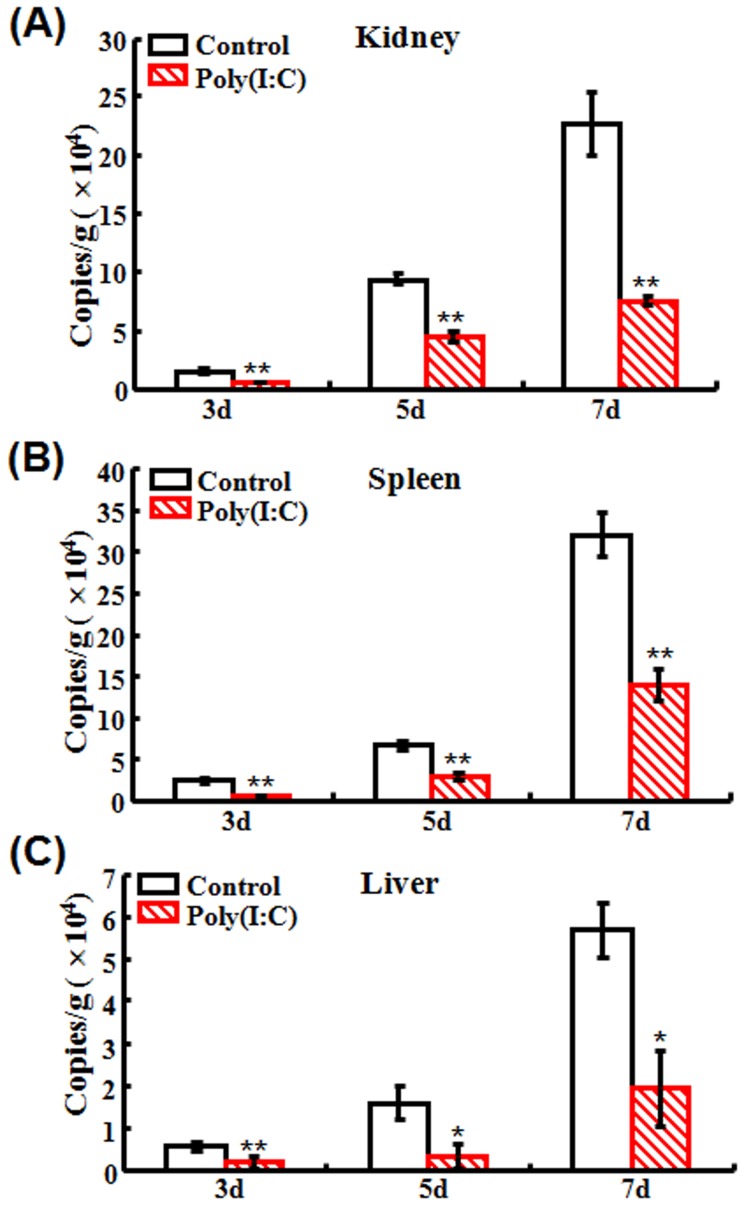
Antiviral effect of poly(I:C) in Japanese flounder. Flounder pre-administered with or without (control) poly(I:C) were infected with megalocytivirus. The amounts of virus in kidney (A), spleen (B) and liver (C) were determined at 3 days (d), 5 d, and 7 d post-infection. Data are expressed as the mean ± SE (*N* = 3). *N*, the number of experimental repeat. *P<0.05; **P<0.01.

To investigate the potential effect of poly(I:C) on viral replication in Japanese flounder, flounder were administered with or without poly(I:C) and then infected with megalocytivirus. At 3 days post-infection (dpi), 5 dpi, and 7 dpi, viral loads in kidney, spleen, and liver of the fish were examined. The results showed that treatment with poly(I:C) significantly reduced the number of virus in all examined tissues at all examined time points (Fig. 1).

### Effect of poly(I:C) on the activity of immune cells

#### (i) Effect on the respiratory burst of HKM

**Figure 2:**
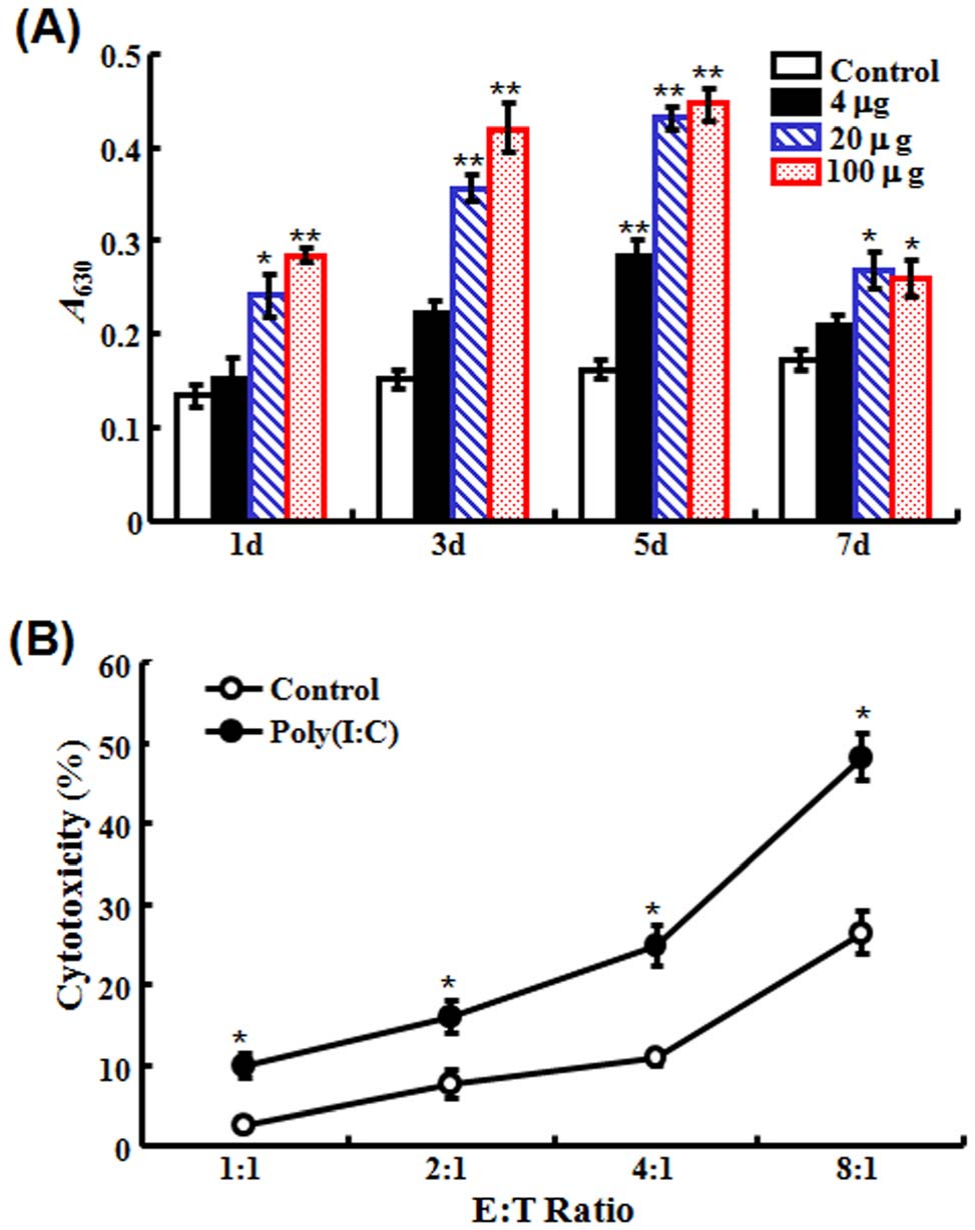
Effect of poly(I:C) on the activation of head kidney macrophages (HKM) and peripheral blood leukocytes (PBL). (A) Flounder were treated with or without (control) different concentrations of poly(I:C); at different days post-treatment, HKM were collected from the fish and examined for respiratory burst activity. (B) PBL collected from flounder treated with or without (control) poly(I:C) were used as effector cells, while PBL collected from megalocytivirus-infected flounder were used as target cells. The effector (E) and target (T) cells were mixed at different ratios and incubated at 22°C for 24 h. The cytotoxicity of the effector cells was then determined by LDH assay. For both panels, data are presented as means ± SE (*N* = 3). *N*, the number of experimental repeat. *P<0.05; **P<0.01.

To examine whether poly(I:C) had any effect on the activation of immune cells, flounder were administered with different concentrations of poly(I:C), and at 1 d, 3 d, 5 d, and 7 d post-administration, HKM were collected from the fish and examined for respiratory burst activity. The results showed that treatment with 20 µg and 100 µg poly(I:C) induced significant and comparable levels of respiratory burst activity at all examined time points, while treatment with 4 µg poly(I:C) induced significant induction of respiratory burst only at 5 d, which in magnitude was much lower than those induced by higher doses of poly(I:C) (Fig. 2A).

#### (ii) Effect on the cytotoxicity of PBL

To examine the effect of poly(I:C) on PBL activation, PBL were collected from flounder pre-treated with or without poly(I:C). In subsequent LDH assay, these PBL served as effector cells and were incubated at different ratios (1∶1, 2∶1, 4∶1, and 8∶1) with target PBL from megalocytivirus-infected flounder. The results showed that pre-treatment with poly(I:C) significantly increased the cytotoxicity of the effector PBL at all effector∶target ratios (Fig. 2B).

### Effect of TLR3 knockdown on poly(I:C)-induced immune response

#### (i) Effect on poly(I:C)-induced gene expression

**Figure 3:**
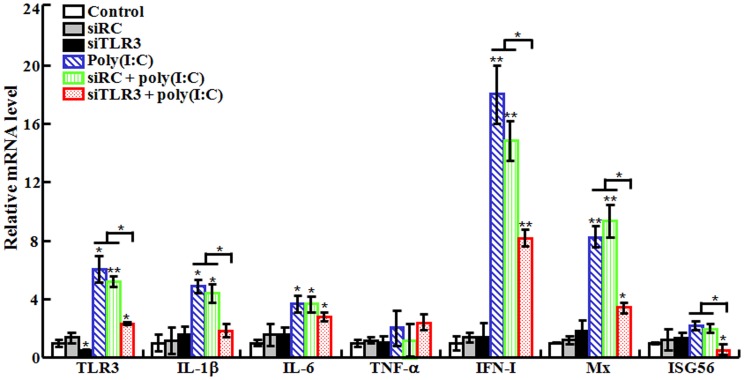
Effect of TLR3 knockdown on poly(I:C)-induced gene expression. Japanese flounder were administered with PBS (control), siRC, siTLR3, poly(I:C), or poly(I:C) in the presence siRC or siTLR3. The expression of immune genes in spleen was then examined by quantitative real time RT-PCR. Data are presented as means ± SE (*N* = 3). *N*, the number of experimental repeat. *P<0.05; **P<0.01.

To examine whether the above observed effect of poly(I:C) was mediated through TLR3 signaling pathway, the expression of TLR3 was knocked down by siRNA. For this purpose, two siRNAs were used, i.e., siTLR3, a TLR3-specific siRNA, and siRC, a nonspecific siRNA. The effect of siTLR3 was determined in flounder FG cells, which showed that in cells transfected with siTLR3, the expression of TLR3 was significantly reduced compared to that in un-transfected cells and in cells transfected with siRC (Fig. S1). To examine the effect of TLR3 knockdown on poly(I:C)-induced gene expression, flounder were pre-administered with siTLR3 or siRC before being treated with poly(I:C), and the expression of immune genes involved in TLR3 signaling pathway was subsequently determined by qRT-PCR. The results showed that in fish treated with siTLR3, TLR3 mRNA level was reduced by 55.7% (Fig. 3), suggesting that siTLR3 effectively interfered with TLR3 expression. In fish treated with poly(I:C) alone and with siRC plus poly(I:C), the expression levels of TLR3, IL-1β, IL-6, IFN-I, and Mx were comparable and significantly higher than those in the untreated control fish (Fig. 3). In contrast, in fish treated with siTLR3 plus poly(I:C), the expression levels of TLR3, IL-1β, IFN-I, Mx, and ISG56 were significantly reduced compared to those in fish treated with poly(I:C) alone.

#### (ii) Effect on poly(I:C)-induced activation of immune cells

**Figure 4:**
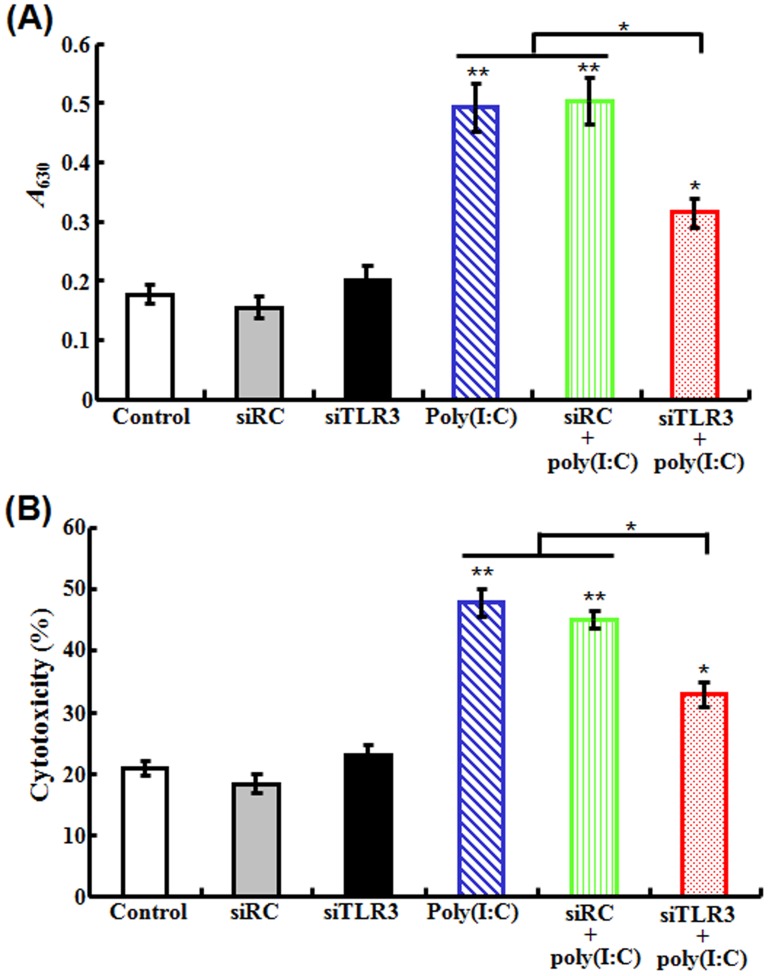
Effect of TLR3 knockdown on poly(I:C)-induced activation of head kidney macrophages (HKM) and peripheral blood leukocytes (PBL). HKM and PBL were collected from flounder treated with PBS (control), siRC, siTLR3, poly(I:C), or poly(I:C) in the presence of siRC or siTLR3. The respiratory burst activity of HKM (A) and the cytotoxicity of PBL (B) were examined. Data are presented as means ± SE (*N* = 3). *N*, the number of experimental repeat. *P<0.05; **P<0.01.

To examine whether TLR3 knockdown affected poly(I:C)-induced activation of immune cells, poly(I:C) was administered into flounder that had been pre-treated with siTLR3 or siRC. HKM of the fish were examined for respiratory burst. The results showed that pre-treatment with siTLR3, but not siRC, significantly inhibited the respiratory burst activity induced by poly(I:C) (Fig. 4A). LDH assay showed that the cytotoxicity of the PBL from the fish treated with poly(I:C) in the presence of siTLR3, but not in the presence of siRC, was significantly lower than that of the PBL from the fish treated with poly(I:C) alone (Fig. 4B).

#### (iii) Effect on poly(I:C)-induced antiviral activity

**Figure 5:**
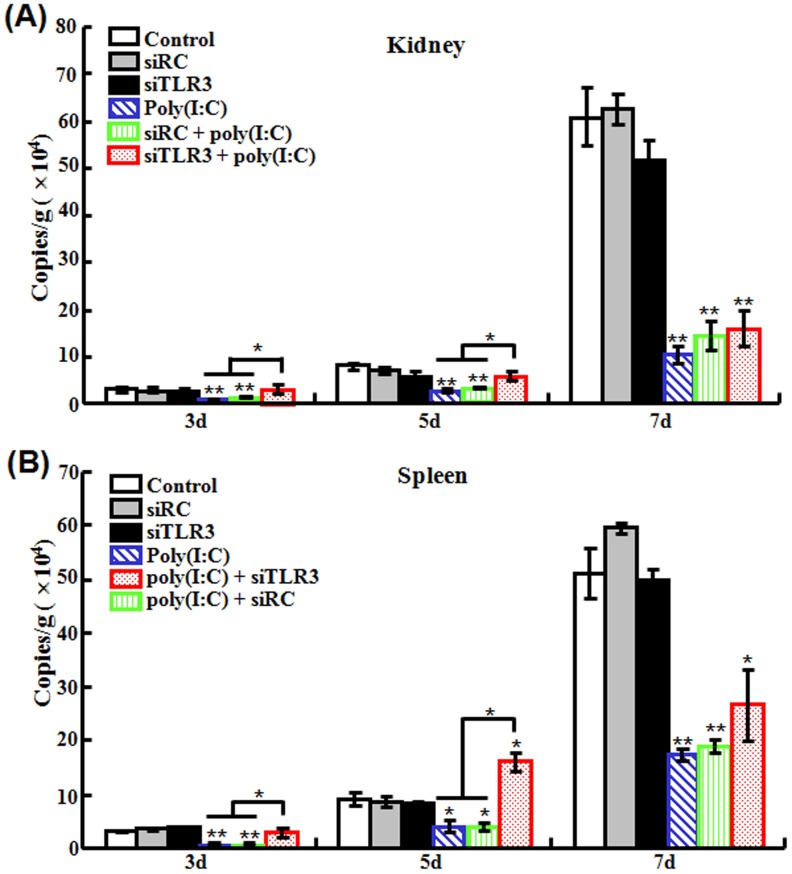
Effect of TLR3 knockdown on poly(I:C)-induced antiviral activity. Japanese flounder were administered with PBS (control), siRC, siTLR3, poly(I:C), or poly(I:C) in the presence siRC or siTLR3. The fish were then infected with megalocytivirus, and the amount of virus in kidney (A) and spleen (B) was determined at different days (d) after infection. Data are expressed as the mean ± SE (*N* = 3). *N*, the number of experimental repeat. *P<0.05; **P<0.01.

To investigate the effect of TLR3 knockdown on poly(I:C)-induced antiviral activity, flounder pre-treated with siTLR3 or siRC were administered with or without poly(I:C). The fish were then infected with megalocytivirus, and the viral loads in kidney and spleen were monitored at 3 dpi, 5 dpi, and 7 dpi. The results showed that in both tissues and at all examined time points, the viral amounts in the fish treated with poly(I:C) and with siRC plus poly(I:C) were comparable and significantly lower than those in the untreated control fish; in contrast, at 3 dpi and 5 dpi, the viral amounts in the fish treated with siTLR3 plus poly(I:C) were significantly higher than those in the control fish and in the fish treated with poly(I:C) alone (Fig. 5).

### Effect of MAD5 knockdown on poly(I:C)-induced immune response

**Figure 6:**
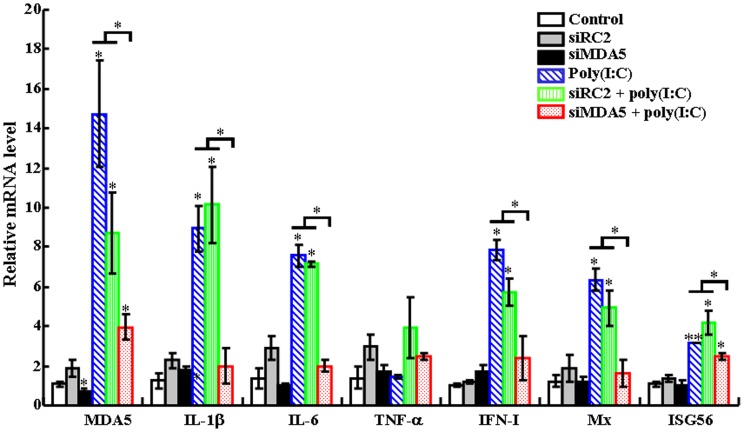
Effect of MDA5 knockdown on poly(I:C)-induced gene expression. Japanese flounder were administered with PBS (control), siRC2, siMDA5, poly(I:C), or poly(I:C) in the presence siRC2 or siMDA5. The expression of immune genes in spleen was then examined by quantitative real time RT-PCR. Data are presented as means ± SE (*N* = 3). *N*, the number of experimental repeat. *P<0.05; **P<0.01.

**Figure 7:**
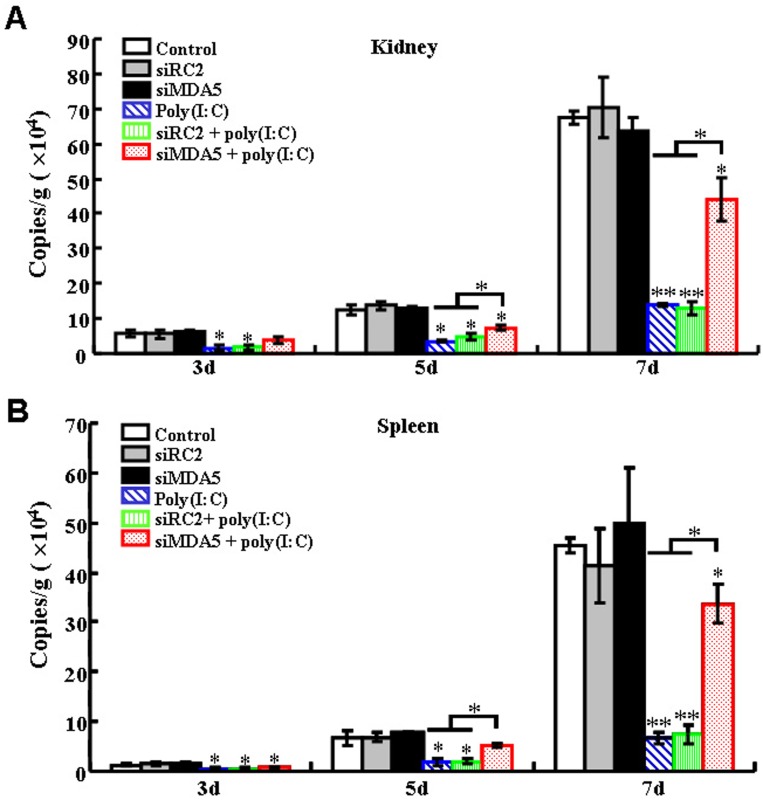
Effect of MDA5 knockdown on poly(I:C)-induced antiviral activity. Japanese flounder were administered with PBS (control), siRC2, siMDA5, poly(I:C), or poly(I:C) in the presence siRC2 or siMDA5. The fish were then infected with megalocytivirus, and the amount of virus in kidney (A) and spleen (B) was determined at different days (d) after infection. Data are expressed as the mean ± SE (*N* = 3). *N*, the number of experimental repeat. *P<0.05; **P<0.01.

Since, as shown above, TLR3 knockdown reduced partly the immune effect of poly(I:C), we further examined whether other factors besides TLR3 were involved in poly(I:C)-induced response. For this purpose, we surveyed the available genes of flounder and selected MDA5 as a target. Three siRNAs targeting MDA5 were examined for interfering efficiency, and one siRNA, siMDA5, was found to be able to significantly reduce the expression of MDA5 after transfection of FG cells (Fig. S2). Consistently, when flounder was administered with siMDA5, MDA5 expression was significantly reduced (Fig. 6). Immune gene expression analysis showed that in flounder treated with siMDA5 plus poly(I:C), the expression levels of IL-1β, IL-6, IFN-I, Mx, and ISG56 were significantly lower than those in fish treated with poly(I:C) alone or with poly(I:C) plus the control siRNA, siRC2 (Fig. 6). Viral infection study showed that when megalocytivirus was inoculated into flounder pre-treated with poly(I:C) or poly(I:C) plus siRC2, the viral loads in kidney and spleen at 3 dpi, 5 dpi, and 7 dpi were significantly lower than those in the untreated control fish (Fig. 7). In contrast, when megalocytivirus was inoculated into flounder pre-treated with siMDA5 plus poly(I:C), the viral loads in kidney were significantly increased at 5 dpi and, especially, 7 dpi compared to those in flounder treated with poly(I:C). Similar results were observed with the viral burdens in spleen.

### Dependence of poly(I:C)-induced immune response on Myd88

#### (i) Effect of Myd88 overexpression on the antiviral activity of poly(I:C)

**Figure 8:**
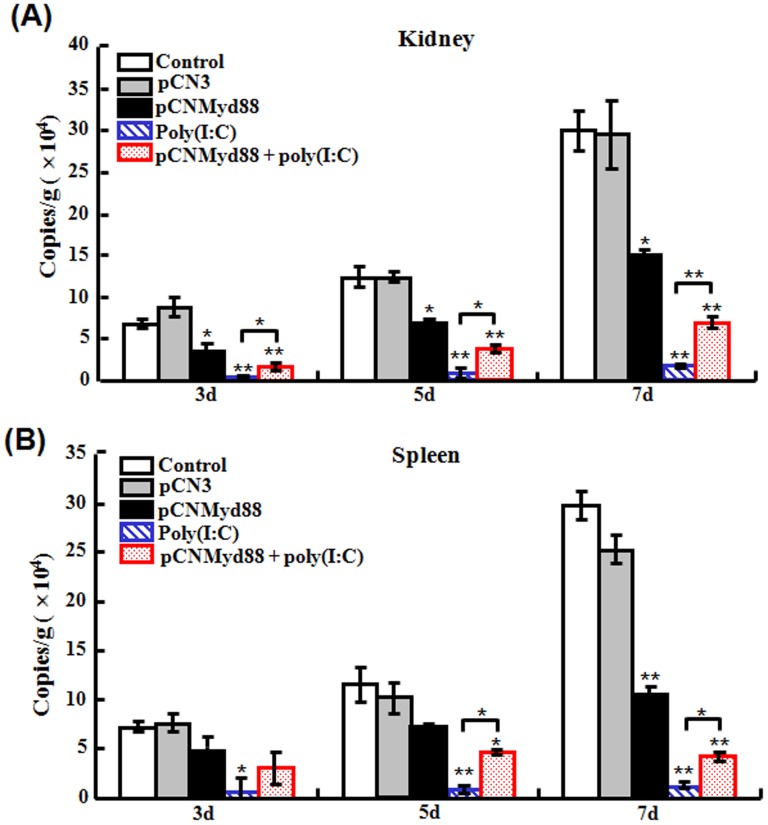
Effect of Myd88 overexpression on the antiviral activity of poly(I:C). Japanese flounder were administered with PBS (control), pCN3, pCNMyd88, poly(I:C), or poly(I:C) in the presence pCNMyd88. The fish were then infected with megalocytivirus, and at different days (d) of infection, the amounts of virus in kidney (A) and spleen (B) were determined. Data are expressed as the mean ± SE (*N* = 3). *N*, the number of experimental repeat. *P<0.05; **P<0.01.

To investigate whether Myd88 contributed to poly(I:C)-induced antiviral activity, overexpression of Myd88 was created in flounder by introducing into the fish the plasmid pCNMyd88, which constitutively expresses flounder Myd88 with a His-tag. Distribution of pCNMyd88 and expression of pCNMyd88-derived Myd88 in fish tissues (muscle, kidney, liver, and spleen) were confirmed by PCR and RT-PCR respectively (Fig. S3 and data not shown). To examine the effect of Myd88 overexpression on poly(I:C)-induced antiviral activity, flounder pre-administered with pCNMyd88 or the control plasmid pCN3 were treated with poly(I:C) and then infected with megalocytivirus. At 3 dpi, 5 dpi, and 7 d pi, the viral loads in kidney and spleen were monitored. The results showed that in kidney, the number of virus in pCNMyd88-treated fish were significantly reduced at all examined time points, however, the reduction folds were much lower than those in poly(I:C)-treated fish (Fig. 8A). In fish treated with pCNMyd88 plus poly(I:C), the viral amounts were significantly higher than those in poly(I:C)-treated fish. Similar results were observed with spleen (Fig. 8B).

#### (ii) Effect of Myd88 inhibition on the antiviral effect of poly(I:C)

**Figure 9:**
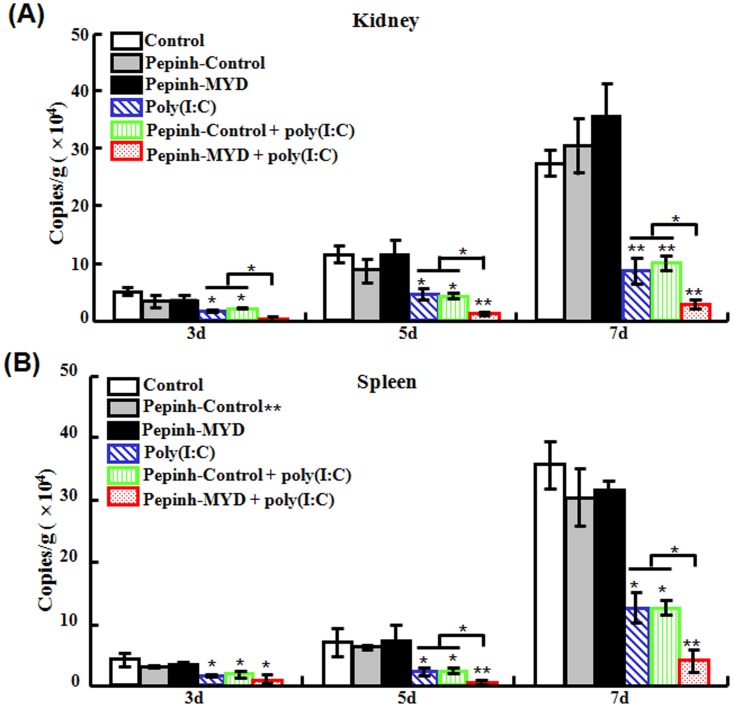
Effect of Myd88 inhibition on the antiviral activity of poly(I:C). Japanese flounder were administered with PBS (control), Pepinh-Control, Pepinh-MYD, poly(I:C), or poly(I:C) in the presence of Pepinh-Control or Pepinh-MYD. The fish were then challenged with megalocytivirus. At different days (d) after challenge, the amounts of virus in kidney (A) and spleen (B) were determined. Data are expressed as the mean ± SE (*N* = 3). *N*, the number of experimental repeat. *P<0.05; **P<0.01.

To further examine the effect of Myd88 on poly(I:C)-induced immune response, flounder were administered with Pepinh-MYD, an inhibitory peptide that blocks Myd88 signaling, or Pepinh-Control, a control peptide, before being treated with poly(I:C). The fish were subsequently infected with megalocytivirus, and at 3 dpi, 5 dpi, and 7 dpi, the viral amounts in kidney and spleen were determined. The results showed that for both tissues, significant reductions of viral number were observed in fish treated with poly(I:C), Pepinh-Control plus poly(I:C), and Pepinh-MYD plus poly(I:C) (Fig. 9). However, in fish treated with Pepinh-MYD plus poly(I:C), the viral amounts were significantly lower than those in fish treated with poly(I:C) alone or with Pepinh-Control plus poly(I:C), the latter two being comparable in levels (Fig. 9).

#### (iii) Effect of Myd88 overexpression and inhibition on poly(I:C)-induced NF-κB activity

**Figure 10:**
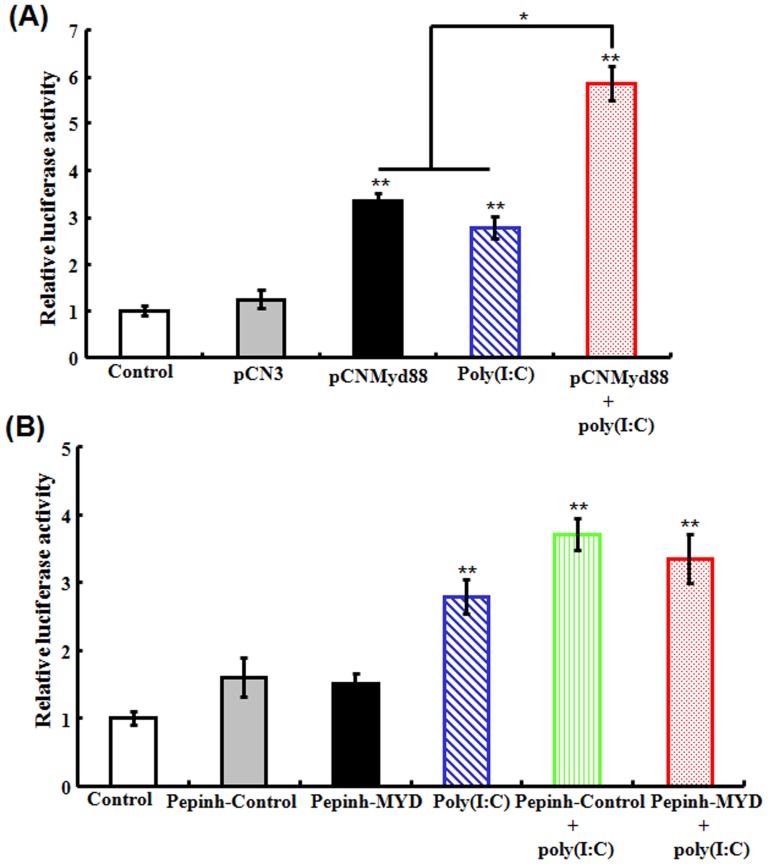
Effect of Myd88 overexpression (A) and inhibition on poly(I:C)-induced NF-κB activity. (A) For Myd88 overexpression, flounder FG-9307 cells containing NF-κB-Luc2 reporter were transfected with or without (control) pCN3 or pCNMyd88. The cells were then treated with or without poly(I:C). (B) For Myd88 inhibition, FG-9307 cells containing NF-κB-Luc2 reporter were treated with or without (control) Pepinh-Control, Pepinh-MYD, poly(I:C), Pepinh-Control plus poly(I:C), or Pepinh-MYD plus poly(I:C). For both panels, the activity of the luciferase reporter was determined at 24 h post-treatment. Data are expressed as the mean ± SE (*N* = 3). *N*, the number of experimental repeat. *P<0.05; **P<0.01.

Our preliminary study showed that poly(I:C) treatment induced elevated NF-κB activity in fish cells. With this observation, we wanted to examine whether this effect of poly(I:C) on NF-κB was dependent on Myd88. For this purpose, a condition of Myd88 overexpression in flounder FG cells containing NF-κB-Luc2, a luciferase reporter of NF-κB activity, was created by introducing into the cells pCNMyd88. The cells were then treated with or without poly(I:C). Subsequent analysis of the luciferase reporter activity showed that in cells transfected with pCNMyd88, the luciferase activity was comparable to that in the untransfected cells treated with poly(I:C) (Fig. 10A). In cells that were both transfected with pCNMyd88 and treated with poly(I:C), the luciferase activity was significantly higher than that in poly(I:C)-treated cells and in pCNMyd88-transfceted cells. To examine the effect of Myd88 inhibition, FG cells containing NF-κB-Luc reporter were treated with poly(I:C) in the presence or absence of Pepinh-MYD or Pepinh-Control. The results showed that the luciferase activities in cells treated with poly(I:C), Pepinh-MYD plus poly(I:C), and Pepinh-Control plus poly(I:C) were comparable (Fig. 10B).

#### (iv) Effect of Myd88 overexpression and inhibition on poly(I:C)-induced gene expression

**Figure 11:**
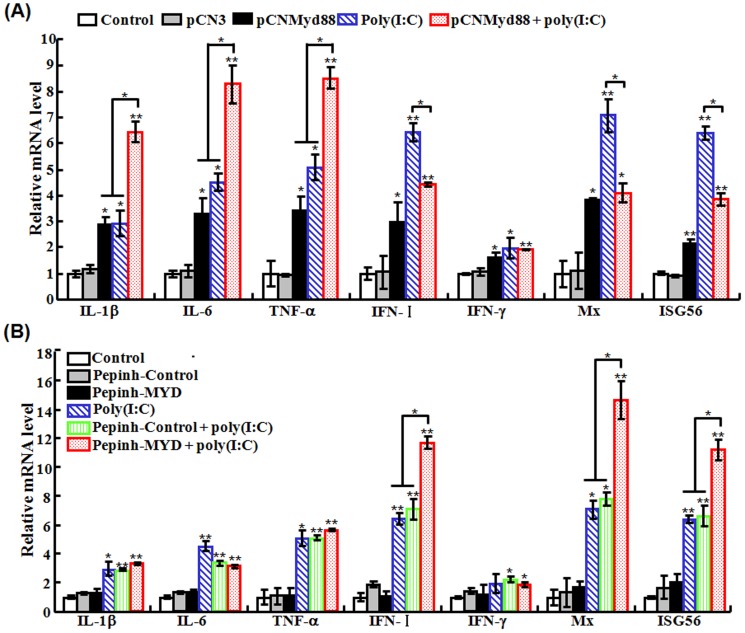
Overexpression (A) and inhibition (B) of Myd88 on the expression of immune-related genes induced by poly(I:C). (A) For Myd88 overexpression, FG-9307 cells were treated with or without (control) pCN3, pCNMyd88, poly(I:C), or pCNMyd88 plus poly(I:C). (B) For Myd88 inhibition, FG-9307 cells were treated with or without (control) Pepinh-Control, Pepinh-MYD, poly(I:C), Pepinh-Control plus poly(I:C), or Pepinh-MYD plus poly(I:C). For both panels, the expression of immune genes was determined by quantitative real time RT-PCR at 24 h post-treatment. Data are expressed as the mean ± SE (*N* = 3). *N*, the number of experimental repeat. *P<0.05; **P<0.01.

qRT-PCR was conducted to determine the mRNA levels of IL-1β, IL-6, TNF-α, IFN-I, IFN-γ, Mx, and ISG56 in FG cells with Myd88 overexpression or inhibition in combination with poly(I:C) treatment as described above. The results showed that in cells transfected with pCNMyd88, the expressions of all genes were significantly upregulated compared to control cells (Fig. 11A). Similar observation was made with cells treated with poly(I:C). In cells transfected with pCNMyd88 and treated with poly(I:C), the expression levels of IL-1β, IL-6, and TNF-α were significantly higher than those in cells transfected with pCNMyd88 or treated with poly(I:C) alone, while the expression levels of IFN-I, Mx, and ISG56 were comparable to those in cells transfected with pCNMyd88 but significantly lower than those in cells treated with poly(I:C). In the case of Myd88 inhibition, qRT-PCR showed that the presence of the Myd88 inhibitor Pepinh-MYD had no effect on poly(I:C)-induced expressions of IL-1β, IL-6, and TNF-α but significantly increased poly(I:C)-induced expressions of IFN-I, Mx, and ISG56 (Fig. 11B).

## Discussion

Previous studies have shown that poly(I:C) exerts antiviral properties in several teleost models [18], [22], [32]–[34]. In this work, we found that in flounder, pre-administration with poly(I:C) before megalocytivirus infection significantly reduced viral replications in kidney, spleen, and liver in a time-dependent manner. This observation, together with those made previously with other teleost species, suggests that poly(I:C) is probably a universal immunostimulant that activates the fundamental antiviral immune response preserved in higher and lower vertebrates. It is known that in mammals, poly(I:C) stimulates the activity of various immune cells, including macrophages [35], DCs [10], NK cells [36], γδT lymphocytes [8], and CTLs [9]. Likewise, in our study, we found that poly(I:C) induced activation of HKM and PBL as reflected by the significantly enhanced respiratory burst activity and cytotoxicity in the presence of poly(I:C).

Conflicting reports on the role of TLR3 pathway in viral infection in humans and mice have been documented. In humans, it has been shown that for some viruses, such as A/H1N1/2009 influenza virus, herpes simplex virus, influenza A virus, hepatitis B virus, and HIV-1, TLR3-mediated immune response is important for protection against viral infection [37]–[43], while for other viruses such as rotavirus, tick-borne encephalitis virus, and hepatitis C virus, TLR3 contributes to a detrimental inflammatory response [44]–[46]. Similarly, in mice, antiviral effects were observed with TLR3 signaling against poliovirus, coxsackievirus B, and encephalomyocarditis virus [47]–[50], whereas host-detrimental effects were observed with TLR3-triggered unbalanced inflammatory response in the infection of rhinovirus type 1B, vaccinia virus, respiratory syncytial virus, and influenza A virus [51]–[54]. In our study, we found that TLR3 knockdown significantly attenuated poly(I:C)-induced expression of immune genes, suggesting that poly(I:C)-mediated immune response is TLR3-dependent. This result is in agreement with a previous report, which showed that stimulation of TLR3-overexpressing fugu cells with poly(I:C) upregulated IFN-β expression [55]. In our study, we also observed that TLR3 knockdown weakened markedly the ability of poly(I:C) to activate HKM and PBLs and, consistently, to suppress viral replication. Together these results indicate a vital contribution of the TLR3 signaling pathway to the antiviral activity of poly(I:C) in flounder.

In mammals, poly(I:C) as an analogue of viral double-stranded RNA (dsRNA) can activate not only TLR3 in the endosome, but also other dsRNA sensors, MDA5, retinoic acid-inducible gene I (RIG-I), and laboratory of genetics and physiology 2 (LGP2), all which belong to the antiviral RIG-I-like receptor (RLR) family in the cytoplasm [56], [57]. Upon poly(I:C) detection, MDA5 and RIG-I transmit signals through the adaptor IFN-β promoter stimulator 1(IPS1), while TLR3 signals through the adaptor TRIF. Both these adaptors initiate downstream signaling pathways that lead to activation of a set of transcription factors, including IFN regulatory factor IRF3/7 and NF-κB, and inductions of type I IFNs, IFN stimulated genes (ISGs), and proinflammatory cytokines [58], [59]. In teleost fish, orthologs of mammalian MDA5 have been identified in Japanese flounder [60], rainbow trout [61], grass carp [62], [63], and zebrafish [64]. It was reported that in fish, MDA5 expression was upregulated in kidney by poly I:C, and overexpressing MDA5 in hirame natural embryo cells induced a lower cytopathic effect against viral hemorrhagic septicemia virus [60], [62]. In addition, MDA5 was shown to be involved in the antiviral immune defense against grass carp reovirus and the spring viraemia of carp virus [63], [64]. In line with these previous reports, in our study, we observed that MDA5 expression in flounder was drastically stimulated by poly(I:C), and that interference with MDA5 expression significantly reduced the ability of poly(I:C) to suppress viral replication. These results indicate that MDA5 plays an indispensable role in the antiviral activity of poly(I:C) in flounder.

For all signaling processes, a balance must be maintained to prevent tissue damage and autoimmunity. In mammalian cells, the TLR3 pathway is negatively regulated by various factors, such as sterile a- and armadillo-motif-containing protein (SARM), receptor-interacting protein 3 (RIP3), zinc-finger protein, and sodium hypophosphite [65]–[68]. Recently, TLR adaptor molecules have also been reported to play a part in the regulation of the TLR3 pathway. For example, the TLR4 adaptor molecule Mal is able to limit TLR3-mediated JNK phosphorylation and TLR3-dependent IFN-β production [69], [70]; Myd88, which is the common downstream adaptor recruited by all TLRs except TLR3 [7], negatively regulates TLR3-induced corneal inflammation [71]. Myd88 has also been shown to inhibit TLR3-dependent IL-6 induction, but not IκB degradation or p38 activation, in murine macrophages [69]. In addition, Myd88 was reported to negatively regulate TLR3-mediated IFN-β and RANTES production, but not TNF-α induction [72]. In our study, we found that the suppressive effect of poly(I:C) on viral replication was significantly decreased in fish overexpressing Myd88 and significantly increased in fish with Myd88 inactivated, suggesting that Myd88 is very likely a negative modulator of poly(I:C)-induced antiviral response in flounder. It is of note that in our study, Myd88 inhibited the expression of IFN-I, Mx and ISG56, which are well-known antiviral components, but had no effect on the expression of the inflammatory cytokines IL-1β, IL-6 and TNF-α induced by poly(I:C). These results suggest the possibility that the operation of Myd88 does not involve NF-κB, which, as in TLR3 signaling, promotes inflammation. This hypothesis is supported by the observation that NF-κB activity induced by poly(I:C) was not affected by Myd88 inhibition. We speculate that IRF3 and/or the downstream IFN-stimulated response element (ISRE), which mediates production of type I IFN in TLR3 signaling in mammals [73], may possibly be affected by Myd88, thus resulting in the enhanced antiviral effect observed in flounder administered with both poly(I:C) and Myd88 inhibitor.

In conclusion, we demonstrate in a model of flounder that poly(I:C) exhibits antiviral property against megalocytivirus mediated, most likely, by TLR3 signaling pathway and MDA5, and that poly(I:C)-mediated immune response is inhibited by Myd88 in a manner that does not impair NF-κB activation. These observations provide new insights to the working mechanisms of poly(I:C), TLR3, and MDA5 in lower vertebrates and indicate for the first time that Myd88 plays a negative regulatory role in TLR signaling in teleost.

## Supporting Information

Figure S1 **Knockdown of TLR3 expression by siRNA.** FG-9307 cells were transfected with siTLR3 or siRC in the transfecting agent Lipo-MAX, and the mRNA levels of TLR3 in the transfectants and in the cells treated with Lipo-MAX or PBS (control) were determined by quantitative real time RT-PCR. For convenience of comparison, the expression level of the control cells was set as 1. Data are presented as means±SE (*N* = 3). ** *P*<0.01.
(TIF)

Figure S2 **Knockdown of MDA5 expression by siRNA.** FG-9307 cells were transfected with siMDA5 or siRC2 in the transfecting agent Lipo-MAX, and the mRNA levels of MDA5 in the transfectants and in the cells treated with Lipo-MAX or PBS (control) were determined by quantitative real time RT-PCR. For convenience of comparison, the expression level of the control cells was set as 1. Data are presented as means±SE (*N* = 3). ** *P*<0.01.
(TIF)

Figure S3 **Detection of pCNMyd8 plasmid (A) and expression of plasmid-derived Myd88 (B) in Japanese flounder tissues.** (A) Flounder were administered with PBS (lanes 1 and 4), pCN3 (lane 2), and pCNMyd88 (lane 3). At 2 days (d) post-administration, DNA was extracted from spleen and used for PCR with primers specific to pCN3 (lanes 1 and 2) and pCNMyd88 (lanes 3 and 4). (B) Flounder were administered with PBS, pCN3, and pCNMyd88 (lanes 1, 2, and 3 respectively). At 2 d post-administration, RNA was extracted from the spleen of the fish and used for RT-PCR with primers specific to plasmid-derived Myd88 (upper panel) or, as an internal control, β-actin (lower panel). M, molecular weight markers.
(TIF)

## References

[1] Yu M, Levine SJ (2011) Toll-like receptor, RIG-I-like receptors and the NLRP3 inflammasome: key modulators of innate immune responses to double-stranded RNA viruses. Cytokine Growth Factor Rev 22: 63–72. 21466970 10.1016/j.cytogfr.2011.02.001 PMC3109132

[2] Perales-Linares R, Navas-Martin S (2013) Toll-like receptor 3 in viral pathogenesis: friend or foe? Immunoloy 140: 153–167. 10.1111/imm.12143 PMC3784162 23909285

[3] Mikami T, Miyashita H, Takatsuka S, Kuroki Y, Matsushima N (2012) Molecular evolution of vertebrate Toll-like receptors: evolutionary rate difference between their leucine-rich repeats and their TIR domains. Gene 503: 235–243. 22587897 10.1016/j.gene.2012.04.007

[4] Matsumoto M, Oshiumi H, Seya T (2011) Antiviral responses induced by the TLR3 pathway. Rev Med Virol 21: 66–67. 10.1002/rmv.680 21312311

[5] Akira S, Takeda K (2004) Toll-like receptor signalling. Nat Rev Immunol 4: 499–511. 15229469 10.1038/nri1391

[6] Oshiumi H, Matsumoto M, Funami K, Akazawa T, Seya T (2003) TICAM-1, an adaptor molecule that participates in Toll-like receptor 3-mediated interferon-beta induction. Nat Immunol 4: 161–167. 12539043 10.1038/ni886

[7] Lee KG, Xu S, Kang ZH, Huo J, Huang M, et al (2012) Bruton's tyrosine kinase phosphorylates Toll-like receptor 3 to initiate antiviral response. Proc Natl Acad Sci U S A109: 5791–5796. 10.1073/pnas.1119238109 PMC3326448 22454496

[8] Shojaei H, Oberg HH, Juricke M, Marischen L, Kunz M, et al (2009) Toll-like receptors 3 and 7 agonists enhance tumor cell lysis by human gammadelta T cells. Cancer Res 69: 8710–8717. 19887600 10.1158/0008-5472.CAN-09-1602

[9] Schulz O, Diebold SS, Chen M, Naslund TI, Nolte MA, et al (2005) Toll-like receptor 3 promotes cross-priming to virus-infected cells. Nature 433: 887–892. 15711573 10.1038/nature03326

[10] Pulko V, Liu X, Krco CJ, Harris KJ, Frigola X, et al (2009) TLR3-stimulated dendritic cells up-regulate B7-H1 expression and influence the magnitude of CD8 T cell responses to tumor vaccination. J Immunol 183: 3634–3641. 19710456 10.4049/jimmunol.0900974 PMC2789393

[11] Hwang SD, Ohtani M, Hikima J, Jung TS, Kondo H, et al (2012) Molecular cloning and characterization of Toll-like receptor 3 in Japanese flounder, *Paralichthys olivaceus* . Dev Comp Immunol 37: 87–96. 22206867 10.1016/j.dci.2011.12.004

[12] Baoprasertkul P, Peatman E, Somridhivej B, Liu Z (2006) Toll-like receptor 3 and TICAM genes in catfish: species-specific expression profiles following infection with *Edwardsiella ictaluri* . Immunogenetics 58: 817–830. 16969679 10.1007/s00251-006-0144-z

[13] Oshiumi H, Tsujita T, Shida K, Matsumoto M, Ikeo K, et al (2003) Prediction of the prototype of the human Toll-like receptor gene family from the pufferfish, *Fugu rubripes*, genome. Immunogenetics 54: 791–800. 12618912 10.1007/s00251-002-0519-8

[14] Rodriguez MF, Wiens GD, Purcell MK, Palti Y (2005) Characterization of Toll-like receptor 3 gene in rainbow trout (*Oncorhynchus mykiss*). Immunogenetics 57: 510–519. 16086174 10.1007/s00251-005-0013-1

[15] Phelan PE, Mellon MT, Kim CH (2005) Functional characterization of full-length TLR3, IRAK-4, and TRAF6 in zebrafish (D*anio rerio*). Mol Immunol 42: 1057–1071. 15829296 10.1016/j.molimm.2004.11.005

[16] Svingerud T, Solstad T, Sun B, Nyrud ML, Kileng O, et al (2012) Atlantic salmon type I IFN subtypes show differences in antiviral activity and cell-dependent expression: evidence for high IFNb/IFNc-producing cells in fish lymphoid tissues. J Immunol 189: 5912–5923. 23169587 10.4049/jimmunol.1201188

[17] Takami I, Kwon SR, Nishizawa T, Yoshimizu M (2010) Protection of Japanese flounder *Paralichthys olivaceus* from viral hemorrhagic septicemia (VHS) by Poly(I:C) immunization. Dis Aquat Organ 89: 109–115. 20402228 10.3354/dao02185

[18] Kim HJ, Oseko N, Nishizawa T, Yoshimizu M (2009) Protection of rainbow trout from infectious hematopoietic necrosis (IHN) by injection of infectious pancreatic necrosis virus (IPNV) or poly(I:C). Dis Aquat Organ 83: 105–113. 19326791 10.3354/dao02000

[19] Jensen I, Albuquerque A, Sommer AI, Robertsen B (2002) Effect of poly I:C on the expression of Mx proteins and resistance against infection by infectious salmon anaemia virus in Atlantic salmon. Fish Shellfish Immunol 13: 311–326. 12443013 10.1006/fsim.2001.0406

[20] Purcell MK, Kurath G, Garver KA, Herwig RP, Winton JR (2004) Quantitative expression profiling of immune response genes in rainbow trout following infectious haematopoietic necrosis virus (IHNV) infection or DNA vaccination. Fish Shellfish Immunol 17: 447–462. 15313511 10.1016/j.fsi.2004.04.017

[21] Lockhart K, Gahlawat SK, Soto-Mosquera D, Bowden TJ, Ellis AE (2004) IPNV carrier Atlantic salmon growers do not express Mx mRNA and poly I:C-induced Mx response does not cure the carrier state. Fish Shellfish Immunol 17: 347–352. 15312661 10.1016/j.fsi.2004.04.011

[22] Plant KP, Harbottle H, Thune RL (2005) Poly I:C induces an antiviral state against Ictalurid Herpesvirus 1 and Mx1 transcription in the channel catfish (*Ictalurus punctatus*). Dev Comp Immunol 29: 627–635. 15784293 10.1016/j.dci.2004.09.008

[23] Wang HR, Hu YH, Zhang WW, Sun L (2009) Construction of an attenuated *Pseudomonas fluorescens* strain and evaluation of its potential as a cross-protective vaccine. Vaccine 27: 4047–4055. 19501788 10.1016/j.vaccine.2009.04.023

[24] Tong SL, Li H, Miao HZ (1997) The establishment and partial characterization ofa continuous fish cell line FG-9307 from the gill of flounder *Paralichthys olivaceus* . Aquaculture 56: 327–333.

[25] Zhang M, Hu YH, Xiao ZZ, Sun Y, Sun L (2012) Construction and analysis of experimental DNA vaccines against megalocytivirus. Fish Shellfish Immunol 33: 1192–1198. 22986024 10.1016/j.fsi.2012.09.010

[26] Yu LP, Hu YH, Sun BG, Sun L (2013) Immunological study of the outer membrane proteins of *Vibrio harveyi*: insights that link immunoprotectivity to interference with bacterial infection. Fish Shellfish Immunol 35: 1293–1300. 23932987 10.1016/j.fsi.2013.07.043

[27] Zhou ZX, Zhang J, Sun L (2014) C7: A CpG oligodeoxynucleotide that induces protective immune response against megalocytivirus in Japanese flounder (*Paralichthys olivaceus*) via toll-like receptor 9-mediated signaling pathway. Dev Comp Immunol 44: 124–132. 24333437 10.1016/j.dci.2013.12.002

[28] Zheng WJ, Sun L (2011) Evaluation of housekeeping genes as references for quantitative real time RT-PCR analysis of gene expression in Japanese flounder (*Paralichthys olivaceus*). Fish Shellfish Immunol 30: 638–645. 21185941 10.1016/j.fsi.2010.12.014

[29] Zhang J, Hu YH, Sun BG, Xiao ZZ, Sun L (2013) Selection of normalization factors for quantitative real time RT-PCR studies in Japanese flounder (*Paralichthys olivaceus*) and turbot (*Scophthalmus maximus*) under conditions of viral infection. Vet Immunol Immunopathol 152: 303–316. 23332581 10.1016/j.vetimm.2012.12.018

[30] Jiao XD, Zhang M, Hu YH, Sun L (2009) Construction and evaluation of DNA vaccines encoding *Edwardsiella tarda* antigens. Vaccine 27: 5195–5202. 19596416 10.1016/j.vaccine.2009.06.071

[31] Chi H, Hu YH, Xiao ZZ, Sun L (2014) Nuclear factor 45 of tongue sole (*Cynoglossus semilaevis*): evidence for functional differentiation between two isoforms in immune defense against viral and bacterial pathogens. Dev Comp Immunol 42: 125–131. 24060504 10.1016/j.dci.2013.09.001

[32] Oh MJ, Takami I, Nishizawa T, Kim WS, Kim CS, et al (2012) Field tests of Poly(I:C) immunization with nervous necrosis virus (NNV) in sevenband grouper, *Epinephelus septemfasciatus* (Thunberg). J Fish Dis 35: 187–191. 22239254 10.1111/j.1365-2761.2011.01334.x

[33] Thim HL, Iliev DB, Christie KE, Villoing S, McLoughlin MF, et al (2012) Immunoprotective activity of a Salmonid Alphavirus Vaccine: comparison of the immune responses induced by inactivated whole virus antigen formulations based on CpG class B oligonucleotides and poly I:C alone or combined with an oil adjuvant. Vaccine 30: 4828–4834. 22634299 10.1016/j.vaccine.2012.05.010

[34] Strandskog G, Villoing S, Iliev DB, Thim HL, Christie KE, et al (2011) Formulations combining CpG containing oliogonucleotides and poly I:C enhance the magnitude of immune responses and protection against pancreas disease in Atlantic salmon. Dev Comp Immunol 35: 1116–1127. 21527278 10.1016/j.dci.2011.03.016

[35] Reimer T, Brcic M, Schweizer M, Jungi TW (2008) poly(I:C) and LPS induce distinct IRF3 and NF-kappaB signaling during type-I IFN and TNF responses in human macrophages. J Leukoc Biol 83: 1249–1257. 18252870 10.1189/jlb.0607412

[36] Akazawa T, Ebihara T, Okuno M, Okuda Y, Shingai M, et al (2007) Antitumor NK activation induced by the Toll-like receptor 3-TICAM-1 (TRIF) pathway in myeloid dendritic cells. Proc Natl Acad Sci U S A 104: 252–257. 17190817 10.1073/pnas.0605978104 PMC1765444

[37] Esposito S, Molteni CG, Giliani S, Mazza C, Scala A, et al (2012) Toll-like receptor 3 gene polymorphisms and severity of pandemic A/H1N1/2009 influenza in otherwise healthy children. Virol J 9: 270. 23151015 10.1186/1743-422X-9-270 PMC3511245

[38] Guo Y, Audry M, Ciancanelli M, Alsina L, Azevedo J, et al (2011) Herpes simplex virus encephalitis in a patient with complete TLR3 deficiency: TLR3 is otherwise redundant in protective immunity. J Exp Med 208: 2083–2098. 21911422 10.1084/jem.20101568 PMC3182056

[39] Hidaka F, Matsuo S, Muta T, Takeshige K, Mizukami T, et al (2006) A missense mutation of the Toll-like receptor 3 gene in a patient with influenza-associated encephalopathy. Clin Immunol 119: 188–194. 16517210 10.1016/j.clim.2006.01.005

[40] Rong Y, Song H, You S, Zhu B, Zang H, et al (2013) Association of Toll-like receptor 3 polymorphisms with chronic hepatitis B and hepatitis B-related acute-on-chronic liver failure. Inflammation 36: 413–418. 23076446 10.1007/s10753-012-9560-4

[41] Li G, Zheng Z (2013) Toll-like receptor 3 genetic variants and susceptibility to hepatocellular carcinoma and HBV-related hepatocellular carcinoma. Tumour Biol 34: 1589–1594. 23404408 10.1007/s13277-013-0689-z

[42] Swaminathan G, Rossi F, Sierra LJ, Gupta A, Navas-Martin S, et al (2012) A role for microRNA-155 modulation in the anti-HIV-1 effects of Toll-like receptor 3 stimulation in macrophages. PLoS Pathog 8: e1002937. 23028330 10.1371/journal.ppat.1002937 PMC3447756

[43] Rivieccio MA, Suh HS, Zhao Y, Zhao ML, Chin KC, et al (2006) TLR3 ligation activates an antiviral response in human fetal astrocytes: a role for viperin/cig5. J Immunol 177: 4735–4741. 16982913 10.4049/jimmunol.177.7.4735

[44] Pott J, Stockinger S, Torow N, Smoczek A, Lindner C, et al (2012) Age-dependent TLR3 expression of the intestinal epithelium contributes to rotavirus susceptibility. PLoS Pathog 8: e1002670. 22570612 10.1371/journal.ppat.1002670 PMC3343008

[45] Kindberg E, Vene S, Mickiene A, Lundkvist A, Lindquist L, et al (2011) A functional Toll-like receptor 3 gene (TLR3) may be a risk factor for tick-borne encephalitis virus (TBEV) infection. J Infect Dis 203: 523–528. 21216866 10.1093/infdis/jiq082 PMC3071239

[46] Wornle M, Schmid H, Banas B, Merkle M, Henger A, et al (2006) Novel role of toll-like receptor 3 in hepatitis C-associated glomerulonephritis. Am J Pathol 168: 370–385. 16436653 10.2353/ajpath.2006.050491 PMC1606499

[47] Abe Y, Fujii K, Nagata N, Takeuchi O, Akira S, et al (2012) The toll-like receptor 3-mediated antiviral response is important for protection against poliovirus infection in poliovirus receptor transgenic mice. J Virol 86: 185–194. 22072781 10.1128/JVI.05245-11 PMC3255933

[48] Negishi H, Osawa T, Ogami K, Ouyang X, Sakaguchi S, et al (2008) A critical link between Toll-like receptor 3 and type II interferon signaling pathways in antiviral innate immunity. Proc Natl Acad Sci U S A 105: 20446–20451. 19074283 10.1073/pnas.0810372105 PMC2629334

[49] Abston ED, Coronado MJ, Bucek A, Bedja D, Shin J, et al (2012) Th2 regulation of viral myocarditis in mice: different roles for TLR3 versus TRIF in progression to chronic disease. Clin Dev Immunol 2012: 129486. 22013485 10.1155/2012/129486 PMC3195533

[50] Hardarson HS, Baker JS, Yang Z, Purevjav E, Huang CH, et al (2007) Toll-like receptor 3 is an essential component of the innate stress response in virus-induced cardiac injury. Am J Physiol Heart Circ Physiol 292: H251–258. 16936008 10.1152/ajpheart.00398.2006

[51] Wang Q, Miller DJ, Bowman ER, Nagarkar DR, Schneider D, et al (2011) MDA5 and TLR3 initiate pro-inflammatory signaling pathways leading to rhinovirus-induced airways inflammation and hyperresponsiveness. PLoS Pathog 7: e1002070. 21637773 10.1371/journal.ppat.1002070 PMC3102730

[52] Hutchens M, Luker KE, Sottile P, Sonstein J, Lukacs NW, et al (2008) TLR3 increases disease morbidity and mortality from vaccinia infection. J Immunol 180: 483–491. 18097050 10.4049/jimmunol.180.1.483 PMC4470388

[53] Groskreutz DJ, Monick MM, Powers LS, Yarovinsky TO, Look DC, et al (2006) Respiratory syncytial virus induces TLR3 protein and protein kinase R, leading to increased double-stranded RNA responsiveness in airway epithelial cells. J Immunol 176: 1733–1740. 16424203 10.4049/jimmunol.176.3.1733

[54] Le Goffic R, Balloy V, Lagranderie M, Alexopoulou L, Escriou N, et al (2006) Detrimental contribution of the Toll-like receptor (TLR)3 to influenza A virus-induced acute pneumonia. PLoS Pathog 2: e53. 16789835 10.1371/journal.ppat.0020053 PMC1475659

[55] Matsuo A, Oshiumi H, Tsujita T, Mitani H, Kasai H, et al (2008) Teleost TLR22 recognizes RNA duplex to induce IFN and protect cells from birnaviruses. J Immunol 181: 3474–3485. 18714020 10.4049/jimmunol.181.5.3474

[56] Hafner AM, Corthesy B, Merkle HP (2013) Particulate formulations for the delivery of poly(I:C) as vaccine adjuvant. Adv Drug Deliv Rev 65: 1386–1399. 23751781 10.1016/j.addr.2013.05.013

[57] Li X, Ranjith-Kumar CT, Brooks MT, Dharmaiah S, Herr AB, et al (2009) The RIG-I-like receptor LGP2 recognizes the termini of double-stranded RNA. J Biol Chem 284: 13881–13891. 19278996 10.1074/jbc.M900818200 PMC2679488

[58] Saito T, Gale M Jr (2008) Differential recognition of double-stranded RNA by RIG-I-like receptors in antiviral immunity. J Exp Med 205: 1523–1527. 18591413 10.1084/jem.20081210 PMC2442628

[59] Sullivan C, Postlethwait JH, Lage CR, Millard PJ, Kim CH (2007) Evidence for evolving Toll-IL-1 receptor-containing adaptor molecule function in vertebrates. J Immunol 178: 4517–4527. 17372010 10.4049/jimmunol.178.7.4517

[60] Ohtani M, Hikima J, Kondo H, Hirono I, Jung TS, et al (2011) Characterization and antiviral function of a cytosolic sensor gene, MDA5, in Japanese flounder, *Paralichthys olivaceus* . Dev Comp Immunol 35: 554–562. 21185857 10.1016/j.dci.2010.12.013

[61] Chang M, Collet B, Nie P, Lester K, Campbell S, et al (2011) Expression and functional characterization of the RIG-I-like receptors MDA5 and LGP2 in Rainbow trout (Oncorhynchus mykiss). J Virol 85: 8403–8412. 21680521 10.1128/JVI.00445-10 PMC3147945

[62] Su J, Huang T, Dong J, Heng J, Zhang R, et al (2010) Molecular cloning and immune responsive expression of MDA5 gene, a pivotal member of the RLR gene family from grass carp Ctenopharyngodon idella. Fish Shellfish Immunol 28: 712–718. 20109556 10.1016/j.fsi.2010.01.009

[63] Chen L, Li Q, Su J, Yang C, Li Y, et al (2013) Trunk kidney of grass carp (*Ctenopharyngodon idella*) mediates immune responses against GCRV and viral/bacterial PAMPs in vivo and in vitro. Fish Shellfish Immunol 34: 909–919. 23333439 10.1016/j.fsi.2013.01.003

[64] Zou PF, Chang MX, Xue NN, Liu XQ, Li JH, et al (2014) Melanoma differentiation-associated gene 5 in zebrafish provoking higher interferon-promoter activity through signalling enhancing of its shorter splicing variant. Immunology 141: 192–202. 24116956 10.1111/imm.12179 PMC3904240

[65] Peng J, Yuan Q, Lin B, Panneerselvam P, Wang X, et al (2010) SARM inhibits both TRIF- and MyD88-mediated AP-1 activation. Eur J Immunol 40: 1738–1747. 20306472 10.1002/eji.200940034

[66] Meylan E, Burns K, Hofmann K, Blancheteau V, Martinon F, et al (2004) RIP1 is an essential mediator of Toll-like receptor 3-induced NF-kappa B activation. Nat Immunol 5: 503–507. 15064760 10.1038/ni1061

[67] Boone DL, Turer EE, Lee EG, Ahmad RC, Wheeler MT, et al (2004) The ubiquitin-modifying enzyme A20 is required for termination of Toll-like receptor responses. Nat Immunol 5: 1052–1060. 15334086 10.1038/ni1110

[68] Yuk JM, Shin DM, Lee HM, Kim JJ, Kim SW, et al (2011) The orphan nuclear receptor SHP acts as a negative regulator in inflammatory signaling triggered by Toll-like receptors. Nat Immunol 12: 742–751. 21725320 10.1038/ni.2064

[69] Kenny EF, Talbot S, Gong M, Golenbock DT, Bryant CE, et al (2009) MyD88 adaptor-like is not essential for TLR2 signaling and inhibits signaling by TLR3. J Immunol 183: 3642–3651. 19717524 10.4049/jimmunol.0901140

[70] Siednienko J, Halle A, Nagpal K, Golenbock DT, Miggin SM (2010) TLR3-mediated IFN-beta gene induction is negatively regulated by the TLR adaptor MyD88 adaptor-like. Eur J Immunol 40: 3150–3160. 20957750 10.1002/eji.201040547

[71] Johnson AC, Li X, Pearlman E (2008) MyD88 functions as a negative regulator of TLR3/TRIF-induced corneal inflammation by inhibiting activation of c-Jun N-terminal kinase. J Biol Chem 283: 3988–3996. 18057004 10.1074/jbc.M707264200

[72] Siednienko J, Gajanayake T, Fitzgerald KA, Moynagh P, Miggin SM (2011) Absence of Myd88 results in enhanced TLR3-dependent phosphorylation of IRF3 and increased IFN-beta and RANTES production. J Immunol 186: 2514–2522. 21248248 10.4049/jimmunol.1003093

[73] Han KJ, Yang Y, Xu LG, Shu HB (2010) Analysis of a TIR-less splice variant of TRIF reveals an unexpected mechanism of TLR3-mediated signaling. J Biol Chem 285: 12543–12550. 20200155 10.1074/jbc.M109.072231 PMC2857105

