# Supplementary figures and images for: Poly(I:C) Induces Antiviral Immune Responses in Japanese Flounder (Paralichthys olivaceus) That Require TLR3 and MDA5 and Is Negatively Regulated by Myd88
Source: PLoS One. 2014 Nov 13;9(11):e112918. doi: 10.1371/journal.pone.0112918 (PMC4231074; doi:10.1371/journal.pone.0112918)

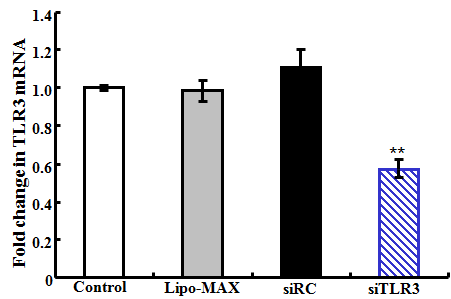

Supplement: Figure S1 — Knockdown of TLR3 expression by siRNA. FG-9307 cells were transfected with siTLR3 or siRC in the transfecting agent Lipo-MAX, and the mRNA levels of TLR3 in the transfectants and in the cells treated with Lipo-MAX or PBS (control) were determined by quantitative real time RT-PCR. For convenience of comparison, the expression level of the control cells was set as 1. Data are presented as means±SE (N = 3). ** P<0.01. (TIF) [file pone.0112918.s001.tif]

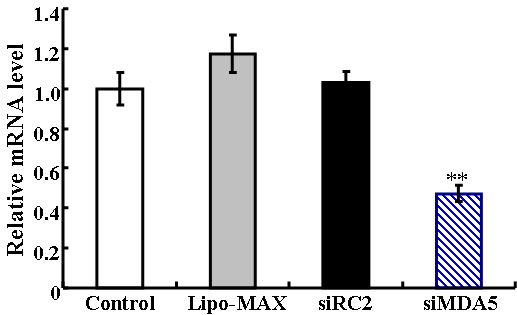

Supplement: Figure S2 — Knockdown of MDA5 expression by siRNA. FG-9307 cells were transfected with siMDA5 or siRC2 in the transfecting agent Lipo-MAX, and the mRNA levels of MDA5 in the transfectants and in the cells treated with Lipo-MAX or PBS (control) were determined by quantitative real time RT-PCR. For convenience of comparison, the expression level of the control cells was set as 1. Data are presented as means±SE (N = 3). ** P<0.01. (TIF) [file pone.0112918.s002.tif]

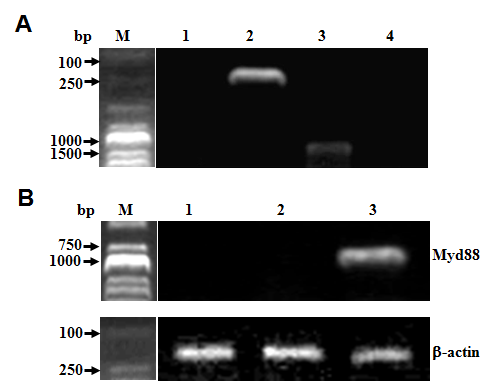

Supplement: Figure S3 — Detection of pCNMyd8 plasmid (A) and expression of plasmid-derived Myd88 (B) in Japanese flounder tissues. (A) Flounder were administered with PBS (lanes 1 and 4), pCN3 (lane 2), and pCNMyd88 (lane 3). At 2 days (d) post-administration, DNA was extracted from spleen and used for PCR with primers specific to pCN3 (lanes 1 and 2) and pCNMyd88 (lanes 3 and 4). (B) Flounder were administered with PBS, pCN3, and pCNMyd88 (lanes 1, 2, and 3 respectively). At 2 d post-administration, RNA was extracted from the spleen of the fish and used for RT-PCR with primers specific to plasmid-derived Myd88 (upper panel) or, as an internal control, β-actin (lower panel). M, molecular weight markers. (TIF) [file pone.0112918.s003.tif]
